# Atypical esophageal submucosal tumor lesion with aortoesophageal fistula after thoracic endovascular aortic repair

**DOI:** 10.1055/a-2229-4347

**Published:** 2024-01-23

**Authors:** Koichi Soga

**Affiliations:** 1 26263 Department of Gastroenterology, Dokkyo Medical University Saitama Medical Center, Koshigaya, Japan; 2 13813 Department of Gastroenterology, Omihachiman Community Medical Center, Oumihachiman, Japan

Aortoesophageal fistula (AEF) is a rare but lethal entity that is difficult to diagnose 1 . Despite the promising efficacy of thoracic endovascular aortic repair (TEVAR), which promotes the clinical use of this procedure, the incidence of AEF after TEVAR (post-TEVAR AEF) has increased, making it a major complication 2 .

A 77-year-old man who had undergone TEVAR 2 years previously was hospitalized for an iliopsoas abscess. He also had intermittent tarry stools and progressive anemia. Upper gastrointestinal endoscopy (UGE) revealed a submucosal tumor (SMT)-like protrusion that included ulcerative lesions in the upper esophagus ( Fig. 1 **a** . Contrast-enhanced computed tomography (CT) imaging revealed extravasation of contrast outside the aortic lumen ( Fig. 1 **b** ). The man’s symptoms were due to the presence of a post-TEVAR AEF accompanied by a stent graft infection; subsequently, a second TEVAR procedure was performed. Seven days postoperatively, UGE revealed an ulcerative lesion without debris ( Fig. 1 **c** ). Two months postoperatively, the contrast-enhanced CT image showed contrast agent in the aortic lumen with no evidence of leakage ( Fig. 1 **d** ).

**Fig. 1:**
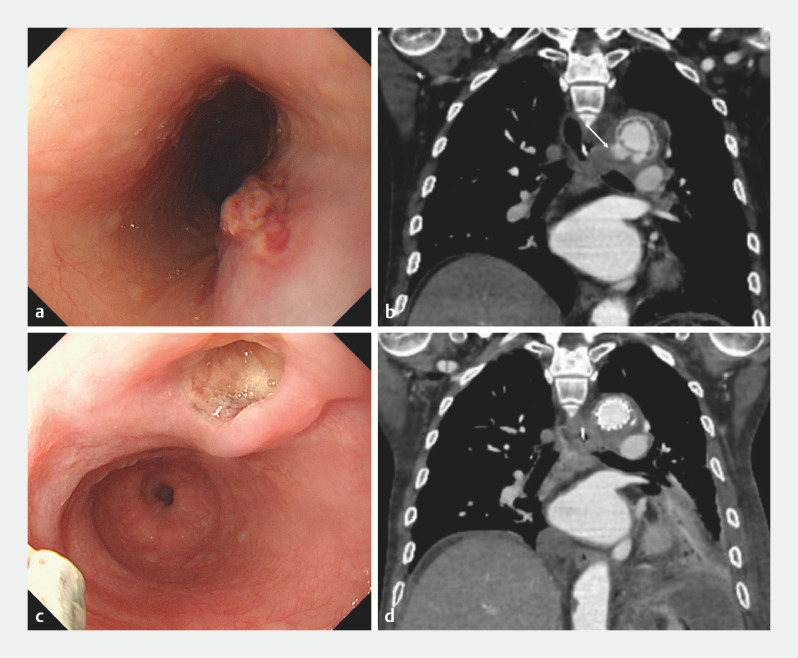
**a, b** Imaging studies in a patient with intermittent tarry stools and progressive anemia 2 years after thoracic endovascular aortic repair (TEVAR). **a** Upper gastrointestinal endoscopy (UGE) reveals a submucosal tumor-like protrusion, including an ulcerative lesion in the upper esophagus. **b** Contrast-enhanced computed tomography (CT) shows contrast extravasation outside the aortic lumen. **c** At 7 days after a second TEVAR, UGE shows an ulcerative lesion without debris. **d** At 2 months after the second TEVAR, contrast-enhanced CT shows the contrast agent entering the aortic lumen with no leakage.

Also at 2 months postoperatively, UGE revealed a recess with an ulcer scar replacing the initial SMT-like lesion (SMTL) ( Fig. 2 **a** ). Two centimeters from the initially detected AEF lesion on the anal side, another SMTL was identified, which had not been found at the first post-TEVAR AEF detection on UGE. The SMTL protruded into and withdrew out of the esophagus in synchronization with breathing ( Fig. 2 **b** ). White-light and narrow-band endoscopic imaging showed that the normal mucosa was elongated with normal vessels near the SMTL ( Video 1 ).

**Fig. 2:**
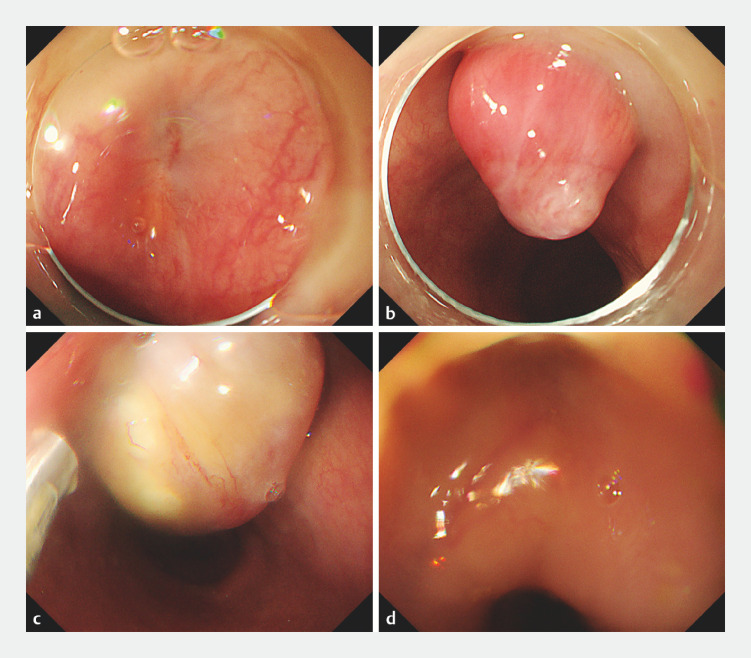
UGE at 2 and 7 months after the second TEVAR. **a, b** After 2 months: **a** UGE shows a recess with an ulcer scar replacing the initial submucosal tumor-like lesion (SMTL) in the upper esophagus (seen in Fig. 1 **a** ). **b** A second SMTL was located 2 cm from the anal side of the initial aortoesophageal fistula (AEF) lesion; this SMTL had not been detected during the initial identification of the AEF. The SMTL repeatedly protruded into and withdrew out of the esophagus in synchronization with breathing. **c, d** After 7 months: **c** UGE now shows an SMTL on the AEF scar, similar to that seen in **b** . **d** Recess with an ulcer scar replacing the second SMTL shown in **b** .

Atypical esophageal submucosal tumor lesion with aortoesophageal fistula after thoracic endovascular aortic repair.Video 1

We suspected that the secondary SMTL originated as a granular mass lesion due to mediastinal infection from the post-TEVAR AEF onto a fragile localized muscular defect 3 4 5 . Seven months later, a similar SMTL was identified at the AEF scar ( Fig. 2 **c, d** ).

In this article we have described a rare endoscopic finding obtained during the long-term follow-up of a post-TEVAR AEF with SMTL showing anomalous movement.

Endoscopy_UCTN_Code_CCL_1AB_2AC_3AG

## Notes

Endoscopy E-Videos https://eref.thieme.de/e-videos
*E-Videos* is an open access online section of the journal *Endoscopy* , reporting on interesting cases and new techniques in gastroenterological endoscopy. All papers include a high-quality video and are published with a Creative Commons CC-BY license. Endoscopy E-Videos qualify for HINARI discounts and waivers and eligibility is automatically checked during the submission process. We grant 100% waivers to articles whose corresponding authors are based in Group A countries and 50% waivers to those who are based in Group B countries as classified by Research4Life (see: https://www.research4life.org/access/eligibility/ ). This section has its own submission website at https://mc.manuscriptcentral.com/e-videos .

## References

[1] Soga K Kitamura R Takenaka S Progressive endoscopic findings in a case of aortoesophageal fistula Dig Endosc 2012 24 290 10.1111/j.1443-1661.2011.01218.x 22725126

[2] Uno K Koike T Takahashi S Management of aorto-esophageal fistula secondary after thoracic endovascular aortic repair: a review of literature Clin J Gastroenterol 2017 10 393 402 10.1007/s12328-017-0762-z 28766283

[3] Tashima T Ohata K Sakai E Perforation during esophageal submucosal dissection resulting from idiopathic partial muscular defect Endoscopy 2016 48 E84 E85 26951474 10.1055/s-0042-102960

[4] Hikichi T Nakamura J Hashimoto M Circumferential esophageal carcinoma with a localized muscle layer defect that caused perforation during endoscopic submucosal dissection Dig Endosc 2019 31 e113 e114 10.1111/den.13501 31602709

[5] Hikichi T Hashimoto M Nakamura J Esophageal localized muscular defect detected immediately after endoscopic submucosal dissection Dig Endosc 2020 32 e126 e127 10.1111/den.13750 32609388

